# Delayed Urethral Stricture Following Remote Electrocution: A Case Report

**DOI:** 10.7759/cureus.105255

**Published:** 2026-03-15

**Authors:** Ali Akjay, Nawfal Ettoumi, Nabil Ettoumi, Najwa Jmil, Sohaib Bidan, Leticia Dominique Nguema, Jihad El Anzaoui

**Affiliations:** 1 Urology, Moulay Ismail Military Hospital, Meknès, MAR; 2 Urology, University Mohammed VI Polytechnic, Ben Guerir, MAR; 3 Urology, Moulay Ismail Military Hospital, University Sidi Mohamed Ben Abdellah, Meknès, MAR

**Keywords:** buccal mucosa graft, bulbar urethra, electrical injury, electrocution, urethral stricture, urethroplasty

## Abstract

Urethral stricture disease is commonly associated with trauma, iatrogenic injury, or inflammatory conditions. Electrical injuries are a rare cause of urological complications, and their association with urethral stricture formation is exceedingly uncommon, with very limited documentation in the literature. We report the case of a 33-year-old male who presented with progressive lower urinary tract symptoms several years after sustaining an accidental electrical injury. Radiological and endoscopic evaluation revealed a long-segment bulbar urethral stricture. The patient underwent augmented anastomotic urethroplasty using a buccal mucosa graft (BMG). The postoperative course was uneventful, and follow-up assessments demonstrated satisfactory urinary flow and no evidence of recurrence. This case demonstrates that remote electrical injury should be considered a potential, albeit rare, cause of urethral stricture disease. Augmented anastomotic urethroplasty with buccal mucosa graft represents an effective treatment option in such complex cases.

## Introduction

Electrical injuries represent a unique form of trauma in which the severity of internal tissue damage may significantly exceed the apparent extent of external burns. The passage of electrical current through the body can result in complex patterns of injury depending on factors such as voltage, current pathway, duration of exposure, and tissue resistance. Unlike conventional thermal burns, electrical trauma frequently causes deep tissue destruction along the path of current flow, affecting muscles, blood vessels, nerves, and internal organs while leaving relatively limited cutaneous findings. As a result, the clinical evaluation of electrical injuries can be challenging, and serious complications may initially be underestimated [1,2].

Electrical injuries account for a small but significant proportion of burn-related admissions and are associated with high morbidity due to multisystem involvement. In addition to cutaneous burns, complications may include cardiac arrhythmias, neurological deficits, musculoskeletal necrosis, and visceral organ damage [3,4]. Importantly, tissue injury may occur at sites distant from the entry and exit points of the current, reflecting the unpredictable pathway of electrical conduction through tissues of varying resistance.

Involvement of the genitourinary tract in electrical trauma is rare and has been only sporadically described in the literature. When present, such injuries may affect the kidneys, bladder, or external genitalia, often in association with severe pelvic or perineal burns. The urethra, a narrow tubular structure lined with delicate mucosa, may be particularly susceptible to ischemic injury, mucosal necrosis, and subsequent fibrosis. These pathological changes can ultimately lead to urethral stricture, which may manifest weeks to months after the initial insult.

Most reported cases of urethral electrical injury are iatrogenic, occurring during endourological procedures such as Transurethral Resection of the Prostate or Transurethral Resection of Bladder Tumor, where inadvertent electrical current may cause thermal damage to the urethral mucosa. However, to our knowledge, urethral stricture resulting from a remote high-voltage electrical injury has not been previously reported in the literature. In this report, we describe a rare case of delayed urethral stricture following electrical trauma and discuss the possible mechanisms underlying this unusual presentation.

## Case presentation

We report the case of a 33-year-old man with no prior medical or urological history who presented with urethral stricture disease following an electrical injury. The patient was electrocuted while at work when a metallic object he was holding came into contact with an electrical cable, resulting in immediate loss of consciousness. He was transported to the hospital by coworkers.

Upon arrival at the emergency department, after regaining consciousness, the patient developed acute urinary retention. Attempts at urethral catheterization were unsuccessful, and a suprapubic catheter was promptly inserted to relieve urinary obstruction.

The patient denied any prior lower urinary tract symptoms, including weak urinary stream, dysuria, urinary tract infections, hematuria, or previous episodes of urinary retention. He also reported no prior urethral instrumentation, pelvic trauma, sexually transmitted infections, or urological surgery. Trauma evaluation at admission revealed no evidence of blunt perineal trauma or straddle injury, and no pelvic fractures or perineal hematoma was documented.

**Figure 1:**
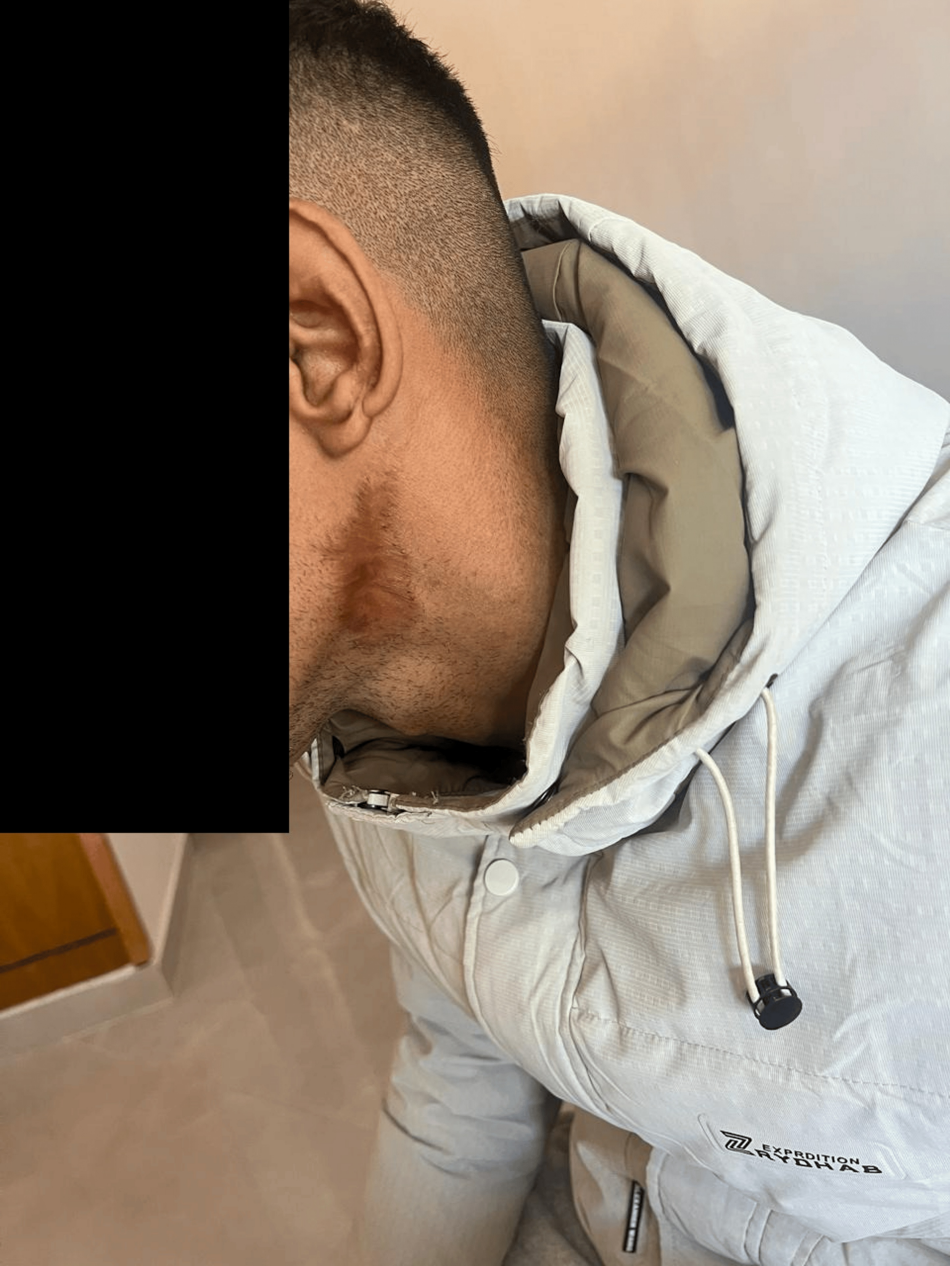
Burning scar on the neck

**Figure 2:**
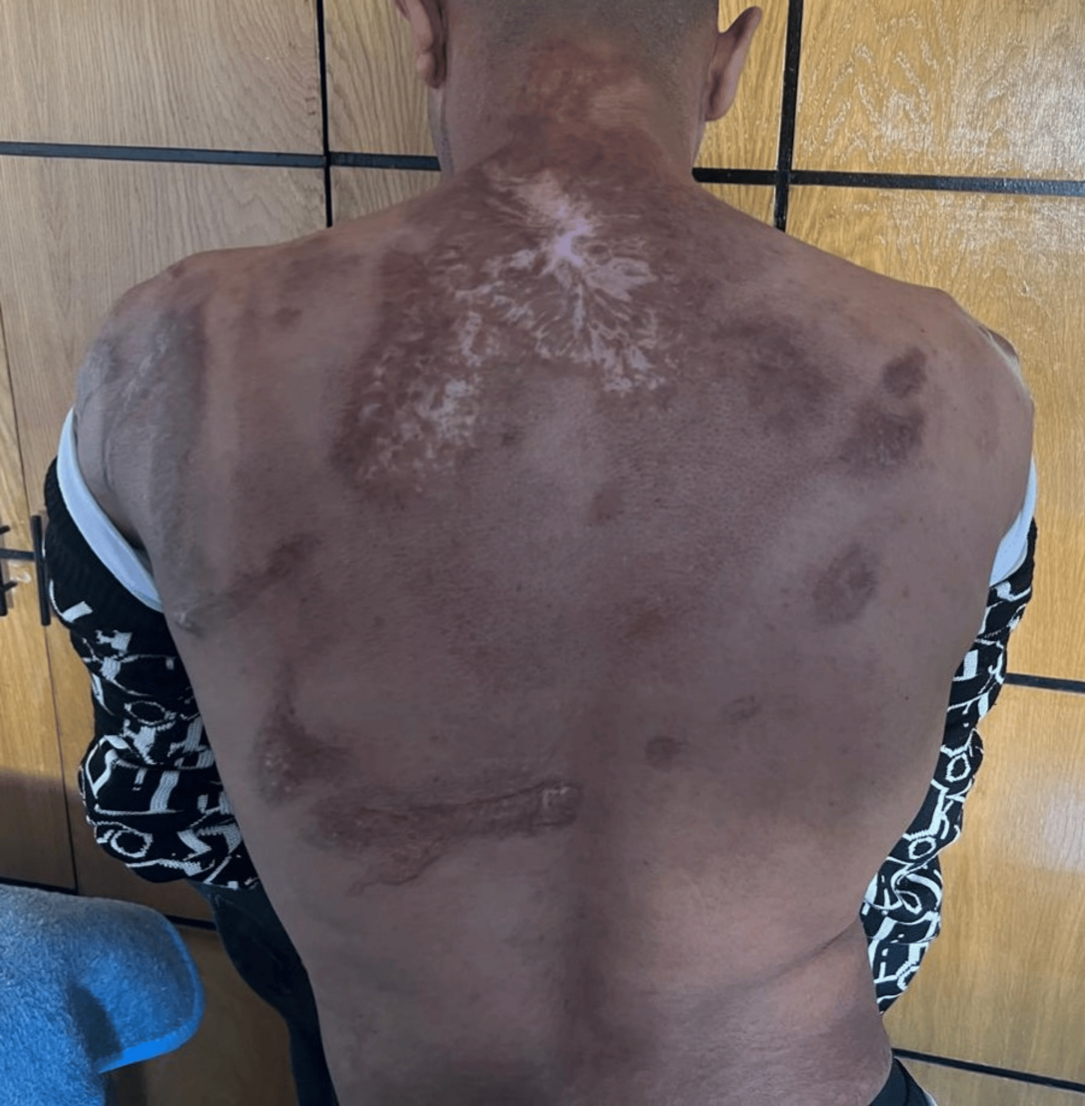
Burning scar on the back

**Figure 3:**
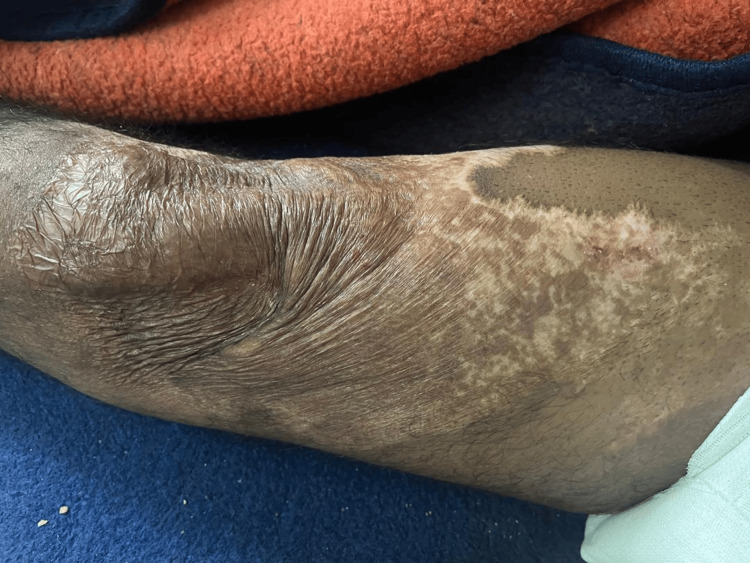
Burning scar on the right leg

The patient also presented with multiple skin burns involving the lateral cervical region of the neck, right leg, and upper back. These lesions were consistent with deep partial-thickness (second-degree) to full-thickness (third-degree) burns, which subsequently healed with scarring (Figures 1-3).

**Figure 4:**
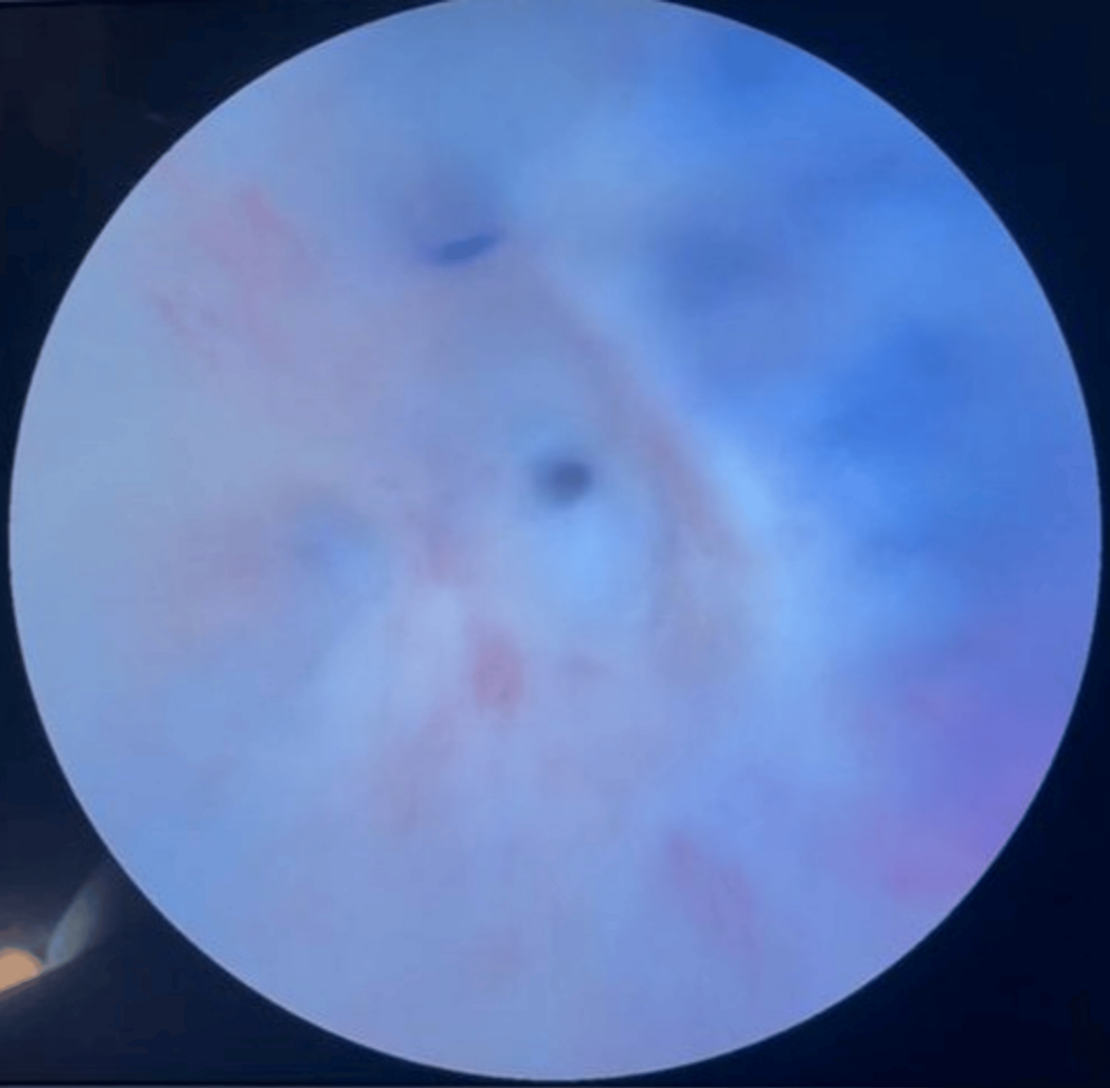
Urethroscopy showing an obliterative urethral stricture

**Figure 5:**
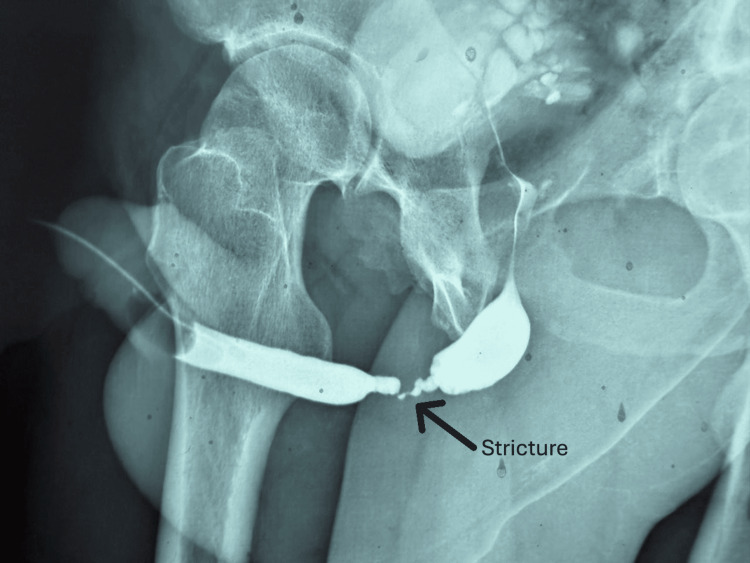
Retrograde uretrogram showing a 4 cm urethral bulbar stricture

During follow-up, urethroscopy (Figure 4) and a retrograde urethrogram (RUG) (Figure 5) were performed. These investigations revealed a long bulbar urethral stricture with a small residual lumen allowing passage of a guidewire.

**Figure 6:**
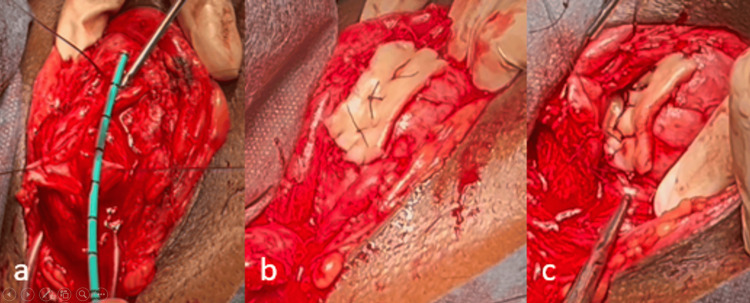
Intraoperative view of augmented anastomotic urethroplasty using buccal mucosa graft (BMG) a: 4 cm long urethral bulbar stricture, b: buccal mucosa graft (BMG) placement, c: anastomosis of both urethral ends with buccal mucosa graft (BMG)

**Figure 7:**
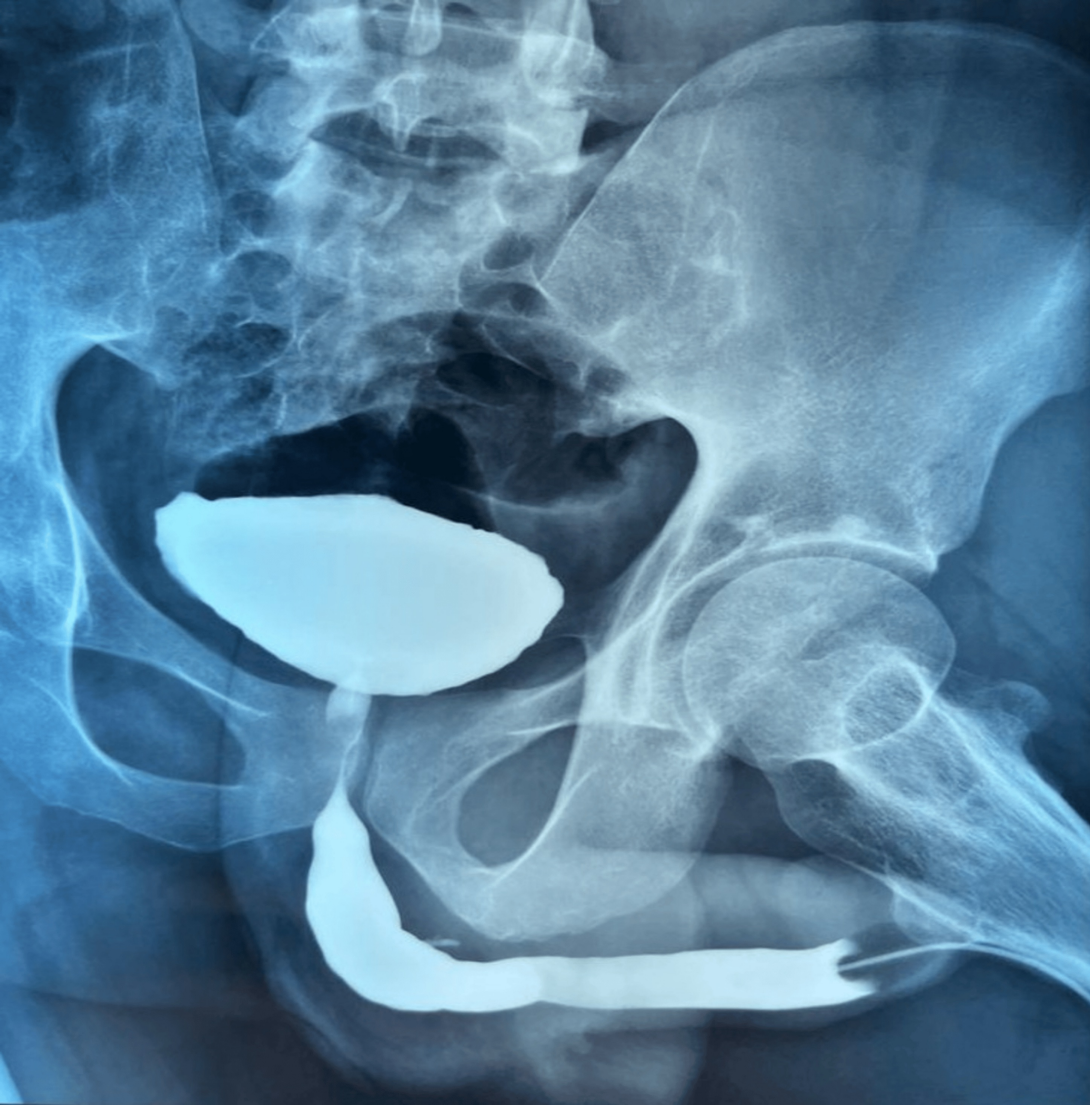
Postoperative retrograde urethrogram (RUG) with no abnormalities

Given the stricture characteristics, surgical reconstruction using augmented anastomotic urethroplasty (Figure 6) was performed with a buccal mucosa graft (BMG) harvested from the inner cheek. Intraoperatively, dense fibrotic tissue was noted at the stricture site, consistent with post-electrical injury fibrosis. A urethral catheter was left in situ for four weeks. Postoperative recovery was uneventful, and at a six-month follow-up with evaluation of post-void residual urine and a retrograde urethrogram (Figure 7), the patient reported normal urinary flow with no recurrence of stricture.

## Discussion

Electrical burns occur when an electric current passes through body tissues, generating thermal energy that results in cellular damage and tissue necrosis. The severity of injury depends on several factors, including the type of current (alternating versus direct), voltage, tissue resistance, duration of exposure, and the pathway of current flow through the body [5].

Remote urethral injury following electrical trauma is believed to occur primarily through vascular and ischemic mechanisms rather than direct contact with the electrical source. The urethral mucosa is highly sensitive to thermal injury, and even brief exposure to high-voltage currents may cause coagulative necrosis. In severe cases, the injury may extend beyond the mucosa to the corpus spongiosum, leading to fibrosis and subsequent urethral stricture formation [6].

Most reported cases of urethral electrical injury are iatrogenic, occurring during endoscopic urological procedures that utilize electrocautery, such as transurethral resection of the prostate (TURP) and transurethral resection of bladder tumor (TURBT). During these procedures, inadvertent thermal damage to the urethral mucosa may occur due to electrical current leakage or prolonged contact with electrosurgical instruments [7,8].

Another rare cause of urethral injury has been described in cases of torture known as “parrilla urethra.” In this practice, electrical wires are wrapped around the penis and sometimes inserted into the urethra to deliver electric shocks. Survivors may develop severe urethral strictures, erectile dysfunction, and significant psychological trauma [9].

To the best of our knowledge, no previous reports have described urethral stricture developing after accidental electrocution without direct urethral contact. In our case, the injury likely resulted from indirect thermal and ischemic damage to the urethral mucosa and the corpus spongiosum, leading to coagulative necrosis followed by progressive fibrosis.

Although the possibility of a previously asymptomatic stricture cannot be entirely excluded, the temporal relationship with the electrical injury and absence of prior symptoms suggest that ischemic and thermal tissue damage along the current pathway may have contributed to the development of the stricture. Because stricture formation may develop weeks to months after the initial injury, clinicians should remain vigilant for urinary symptoms in patients with a history of electrical trauma.

## Conclusions

Urethral stricture may represent a rare and underrecognized delayed complication of accidental electrical injury. Although electrical trauma is primarily associated with cutaneous and musculoskeletal damage, internal tissue injury along the current pathway can lead to progressive fibrosis and late-onset urethral obstruction. This case highlights the importance of considering genitourinary involvement in patients presenting with urinary symptoms after electrical exposure. Careful clinical evaluation, supported by appropriate imaging and endoscopic assessment, is essential for timely diagnosis. When identified, surgical management can successfully restore urinary function and minimize the risk of long-term complications.
